# Oral health care activities performed by caregivers for institutionalized elderly in Barcelona-Spain

**DOI:** 10.4317/medoral.18767

**Published:** 2013-03-25

**Authors:** Marco Cornejo-Ovalle, Kenio Costa-de-Lima, Glória Pérez, Carme Borrell, Elías Casals-Peidro

**Affiliations:** 1 DDS, MSc in Public Health. Faculty of Dentistry, University of Chile - Service of Health Information Systems. Agència de Salut Pública de Barcelona, Spain; 2 MDr, PhD. Agència de Salut Pública de Barcelona - Department of Experimental and Health Sciences. Universitat Pompeu Fabra - CIBER Epidemiología y Salud Pública (CIBERESP), Spain) - Biomedical Research Institute Sant Pau. Barcelona, Spain; 3 DDS, PhD. Federal University of Río Grande do Norte (UFRN). Brasil; 4 DDS, PhD. Primary Health Care Center Sant Miquel, Barcelona, Spain This study is part of the PhD thesis of Marco Cornejo Ovalle at the University Pompeu Fabra in Barcelona

## Abstract

Objectives: To describe the frequency of brushing teeth and cleaning of dentures, performed by caregivers, for institutionalized elderly people. Methods: A cross-sectional study in a sample of 196 caregivers of 31 health centers in Barcelona. The dependent variables were frequency of dental brushing and frequency of cleaning of dentures of the elderly by caregivers. The independent variables were characteristics of caregivers and institutions. We performed bivariate and multivariate descriptive analyses. Robust Poisson regression models were fitted to determine factors associated with the dependent variables and to assess the strength of the association. Results: 83% of caregivers were women, 79% worked on more than one shift, 42% worked only out of necessity, 92% were trained to care for elderly persons, 67% were trained in oral hygiene care for the elderly, and 73% recognized the existence of institutional protocols on oral health among residents. The variables explaining the lower frequency of brushing teeth by caregivers for the elderly, adjusted for the workload, were: no training in the care of elderly persons (PRa 1.7 CI95%: 1.6-1.8), not fully agreeing with the importance of oral health care of the elderly (PRa 2.5 CI95%: 1.5-4.1) and not knowing of the existence of oral health protocols (PRa 1.8 CI95%: 1.2-2.6). The variables that explain the lower frequency of cleaning dentures, adjusted for the workload, were lack of training in elderly care (PRa 1.7 CI95%: 1.3-1.9) and not knowing of the existence of protocols (PRa 3.7 CI95%: 1.6-8.7). Conclusion: The majority of caregivers perform activities of oral health care for the elderly at least once per day. The frequency of this care depends mainly on whether caregivers are trained to perform these activities, the importance given to oral health, the workload of caregivers and the existence of institutional protocols on oral health of institutionalized elderly persons.

** Key words:**Institutionalized elderly, caregivers, oral hygiene, long-term care, oral health.

## Introduction

The demographic transformation of society seen during the past few decades, characterized by a higher proportion of older people, is implying major changes and challenges for health systems and social policy (1). In this context, the increased life expectancy is not always accompanied by a better quality of life (2), because aging can mean some people need help them in performing activities that once seemed simple, so the attention to the needs of dependents is one of the great challenges of social policy (3). This need arises from the act of caring for elderly persons, an interactive process carried out either by informal caregivers who work in the community or in institutions with formal caregivers, whose functions are to ensure oral care, with the aim of helping to maintain the overall health and facilitate the social reintegration of the individual cared for (4,5).

As the percentage of institutionalized elderly persons increases, so does the need for medical and dental care in this population, because the elderly are living longer with natural teeth in the mouth, or using prosthetic devices, thereby imposing greater de-mands for health care in general, and oral health in particular, on the institutionalized population’s caregivers (6). There is evi-dence that clearly links poor oral health with the development of preventable and debilitating systemic conditions that threaten the quality of life and life itself and, therefore, needs to be maintained competently by those who are responsible for health care (7), particularly important for a growing population who are dependent on others for their care. Therefore, the objective need of dental care to avoid conditions that can be reduced with highly cost-effective interventions and adequate oral health care (8), is even greater than before, as diseases that damage such structures are affecting them more than in the past (9).

Previous studies have shown that caregivers of institutionalized elderly consider oral care activities for the elderly as an unplea-sant task (4,10) and empirical observations indicate that this task is usually performed in an inappropriate way (11). For the success of oral care of the elderly it is crucial that caregivers be motivated to devote due attention to oral health (12), because they have a role in encouraging and enabling older people to perform their own oral hygiene, in order to exercise coordination and increase self-esteem (13). Furthermore, caregivers should have good knowledge about proper oral hygiene and proper use of dentures, as these are key to maintaining oral health of elderly people (14). However, oral care of the elderly also depends on the existence of guidelines from the center and on caregivers having time to perform the task (15).

Often the work of caregivers has little visibility, the occupation is often underrated and they rarely have opportunities for training or it is not provided with the required frequency (16). Currently, little is known about the profile of caregivers of the elderly, their needs, their training and performance in relation to the activities of oral health of the institutionalized elderly, a relevant aspect since they play a fundamental role in the daily provision of such care. Accordingly, and based on the conceptual framework proposed by Chami et al. (17) which recognizes the importance not only of the characteristics of caregivers but also the institutional attributes among the factors that hinder the provision of oral hygiene care to institutionalized elderly dependents, the objectives of this study were to describe the specific activities of oral health care (brushing teeth and cleaning dentures) of the elderly performed by caregivers in social-health centers (SHC) with residential activity in the Barcelona Health Region in 2009.

## Material and Methods

-Design, study population and sample: This cross-sectional study was carried out in long stay SHC in the Barcelona Health Region.

The study population was caregivers of institutionalized elderly in the 46 SHC with residential profile belonging to the Barcelona Health Region, these centers together offering 3,876 places in 2009. The exclusion criteria were that caregivers were working in SHC that care for psychiatric patients or that did not agree participating in the study.

The sample size was based on the expected ratio of 1 nursing assistant caregiver for 10 places in the long stay SHC, thus it was verified that there were around 388 caregivers working in shifts, morning and afternoon, and half of those on the night shift, with a total of 582 nursing assistants. For the 46 centers, we estimated 12 nursing assistants for each residency. It was decided to administer the questionnaire to 50% of nursing assistants in each SHC, i.e. 6 per center. A total of 33 SHC agreed to participate in the investigation, 2 of them were excluded for being long-stay centers for psychiatric patients and the final sample size was 196 auxiliary caregivers. The final sample consisted of twice as many day shift (morning or afternoon) caregivers as night shift caregivers. This is because in the SHC there are half the number of caregivers on duty at night, compared to the numbers on duty during the two day shifts, and we aimed to maintain these proportions.

The nursing assistants interviewed were selected from among those who happened to be working at the center on the day of data collection. Those who worked on the night shift were interviewed either immediately after work, or in the evening just before starting their workday-shift.

The coordinators of caregivers explained the data collection method and the people to participate in the study were randomly selected in each SHC. All participants were instructed about the study, signed informed consent and answered a questionnaire specifically designed for this study. The study was approved by the Institutional Ethical Review Board of the Hospital del Mar (CEIC-PSMAR).

-Variables.

The dependent variables were the frequency of performing the activities of brushing teeth and of cleaning dentures of seniors (`more than once a day´, `once a day´, `more than once a month´, `once a month´, `not done´). To evaluate the factors associated with the frequency of the two dependent variables, they were categorized as `≥ once a day´ and `< once a day´, based on recom-mendations available in the literature (18,19) and on existing protocols in Spain ( Table 1).

**Table 1:**
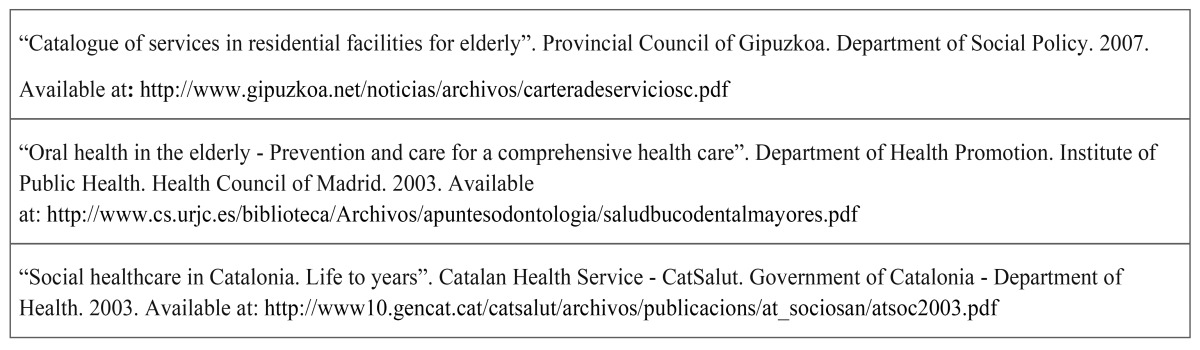
Normative documents or protocols whose portfolio of benefits explicitly mentioned the frequency of brushing teeth for institutionalized elderly people in long-stay residential centers in the Spanish context by 2009.

Independent variables collected were age (stratified according to the median into `16-40 years´ and `41-65 years´), sex, natio-nality (`Spanish´ or `foreign´), educational level (`primary or secondary studies´ and `university or other´), workload (if care-givers work more than one shift), work motivation (`affinity and necessity´, `only need´), nursing assistant training, trained in general health care of elderly, trained in general hygiene of older people, trained in oral hygiene care for the elderly, the im-portance that caregivers put on the care of their own oral health, the importance that caregivers put on the care of the oral health of the elderly (`strongly agree´ Yes / No), experience working with older people (according to median `1-8 years´ , `> 9 year´) and seniority in the institution (according to median `1-4 years´, `> 5 years´). Apart from collecting information regarding the institution’s competence in oral health care for the elderly, such as the existence of protocols for oral health care (Yes / No), we also recorded information from the perspective of caregivers, consulting with their views on whether these protocols were met (Yes / No) and on the availability of oral hygiene items ( Table 2).

**Table 2:**
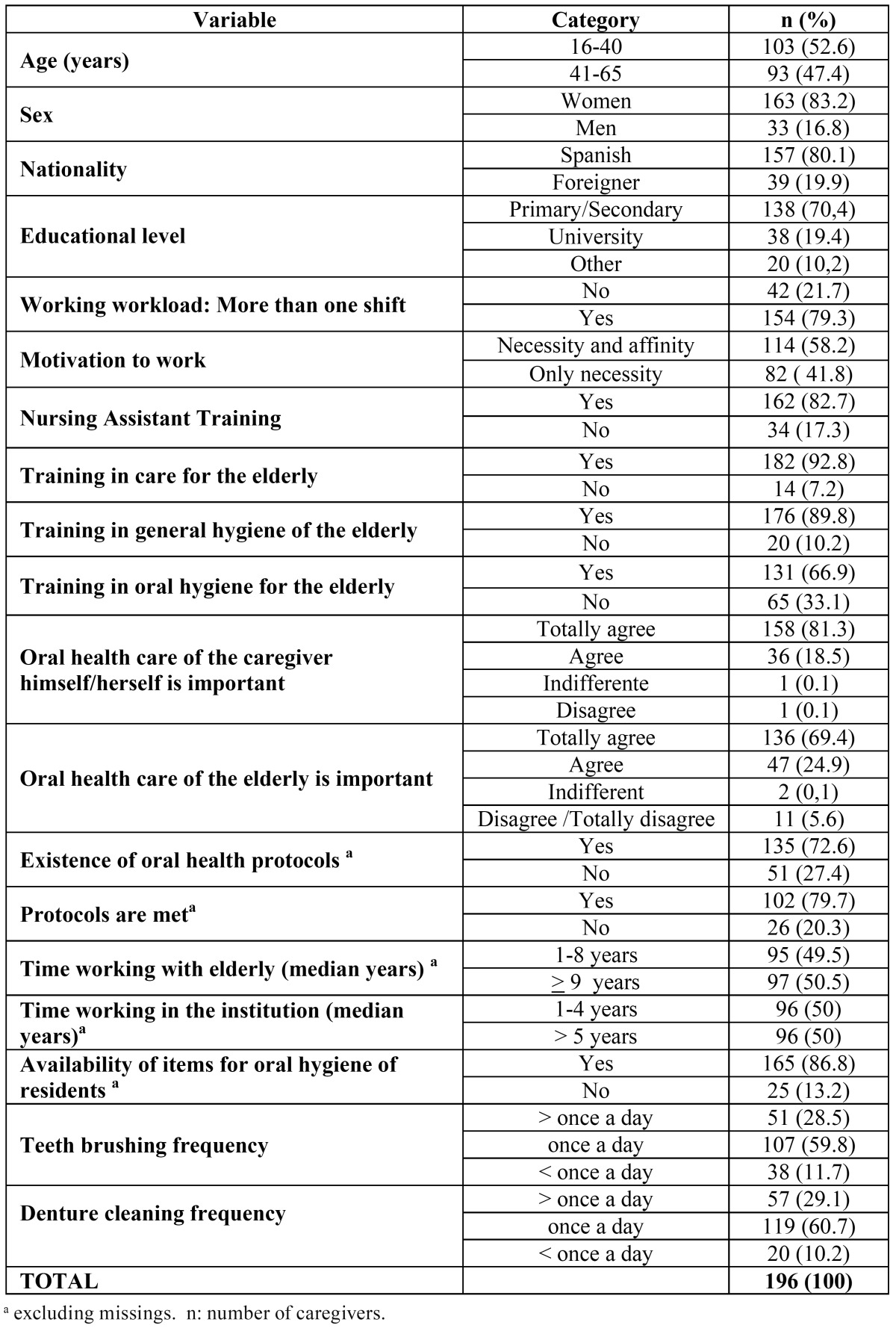
Description of the characteristics of caregivers of institutionalized elderly residents. Social and health centers with residential profile of the Health Region of Barcelona - 2009.

-Data analysis: In addition to descriptive analysis, bivariate analyses were performed using the Pearson chi-square tests to consider clustering (residences). Log-binomial regression was performed and, after evaluating the co linearity between the independent variables using correlation coefficients, multivariate models were fitted to determine the factors associated with the frequency of brushing teeth and cleaning of dentures performed for the elderly by caregivers, and to assess the strength of the association, prevalence ratios (PR) and their confidence intervals at 95% (CI95%) were obtained. The models included variables that were significant according to the test for association (p value <0.05), and also “workload” since this variable influences the degree of opportunities to perform oral care activities for the elderly.

Statistical analyses were carried out using STATA 10 software for Windows.

## Results

Among caregivers there was a higher prevalence of females, of young adults, with a `primary or secondary´ level of studies, most were of Spanish nationality and about 80% worked more than one shift ( Table 2). Regarding the main motivation for work, 42.1% reported that they worked as caregiver for the elderly only out of necessity.

While 82.7% had training as nursing assistant, only about 60% had completed a formal course. Most caregivers were trained to care for the elderly (92.8%) and to take care of general hygiene of these (89.8%), but only 66.9% had training for oral hygiene and denture cleaning in older people. More than 50% had 9 or more years’ experience in care of the elderly.

Although 81% of caregivers strongly agreed with the importance of caring for their own oral health, only 69.4% strongly agreed with the importance of oral health care for the elderly. Regarding the protocols for oral health care for the elderly, 135 caregivers (72.6%) answered that the institution had this kind of guidance. Of these, 75.5% believed that these protocols were met. 86.8% said they had availability of materials for performing oral hygiene and dentures cleaning for the elderly.

In relation to the frequency of brushing teeth and cleaning of oral dental prostheses performed for the elderly, the most frequent response was `only once daily´(59.8% and 60.7% respectively) while 11.7% and 10.2%, respectively, reported doing so `less than once per day´.

As may be seen in  Table 3, the frequency of brushing teeth was significantly associated with caregivers having training in care of elderly persons, training in oral hygiene and cleaning dental prostheses for the elderly, the importance put on their own oral health care and the importance put on the oral health care in the elderly, knowing of the existence of institutional protocols on oral health of residents and whether these protocols were met. Meanwhile, the frequency of cleaning dentures was significantly associated with caregivers being trained to care for the elderly and whether caregivers knew of the existence of institutional protocols on oral health of residents.

**Table 3:**
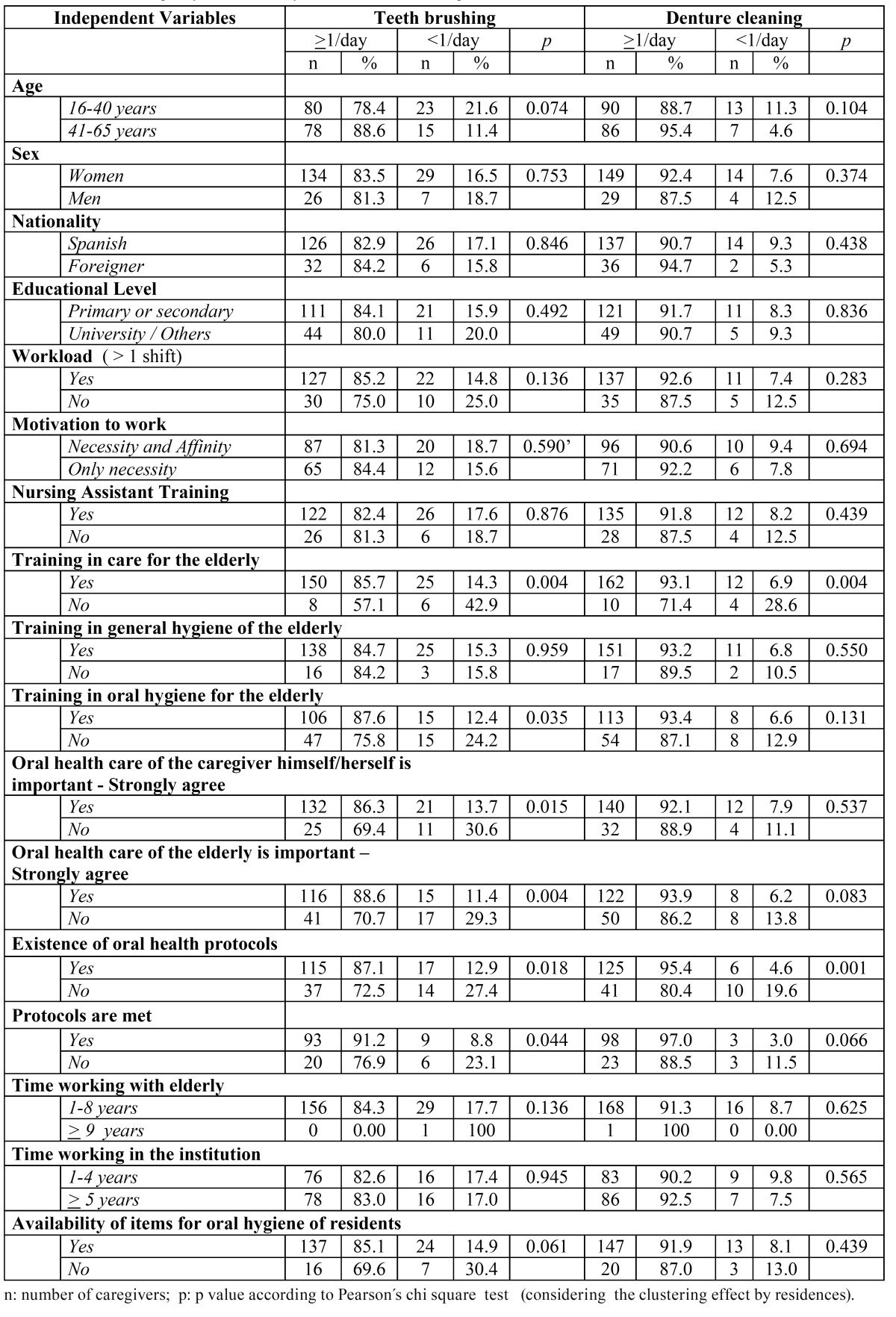
Frequency of the activities of brushing teeth and cleaning dentures performed by caregivers for people 65 years and older institutionalized in long-stay residences, by characteristics of caregivers, Barcelona 2009.

Analysis of the variables that explain the lower frequency of activities of oral hygiene for the elderly ( Table 4), showed that, after adjustment for workload (PRa 1.93; CI95%: 1.33-2.78), those caregivers who brushed the teeth of elderly less frequently than `once per day´ lacked training in care for the elderly (PRa 1.71; CI95%: 1.56- 1.81), did not fully agree with the importance of oral health care for the elderly (PRa 2.47 CI95%: 1.48- 4.10) and knew of the existence of institutional protocols on oral health of residents (PRa 1.80 CI95% 1.24-2.60). Meanwhile, after adjustment for workload, caregivers who performed cleaning of the prosthesis less often (less than once a day) lacked training in caring for the elderly (PRa 1.74; CI 95%: 1.28-1.91 ) and were unaware of the existence of institutional protocols on the oral health of the elderly (PRa 3.74; CI95%: 1.60-8.72) ( Table 4).

**Table 4:**
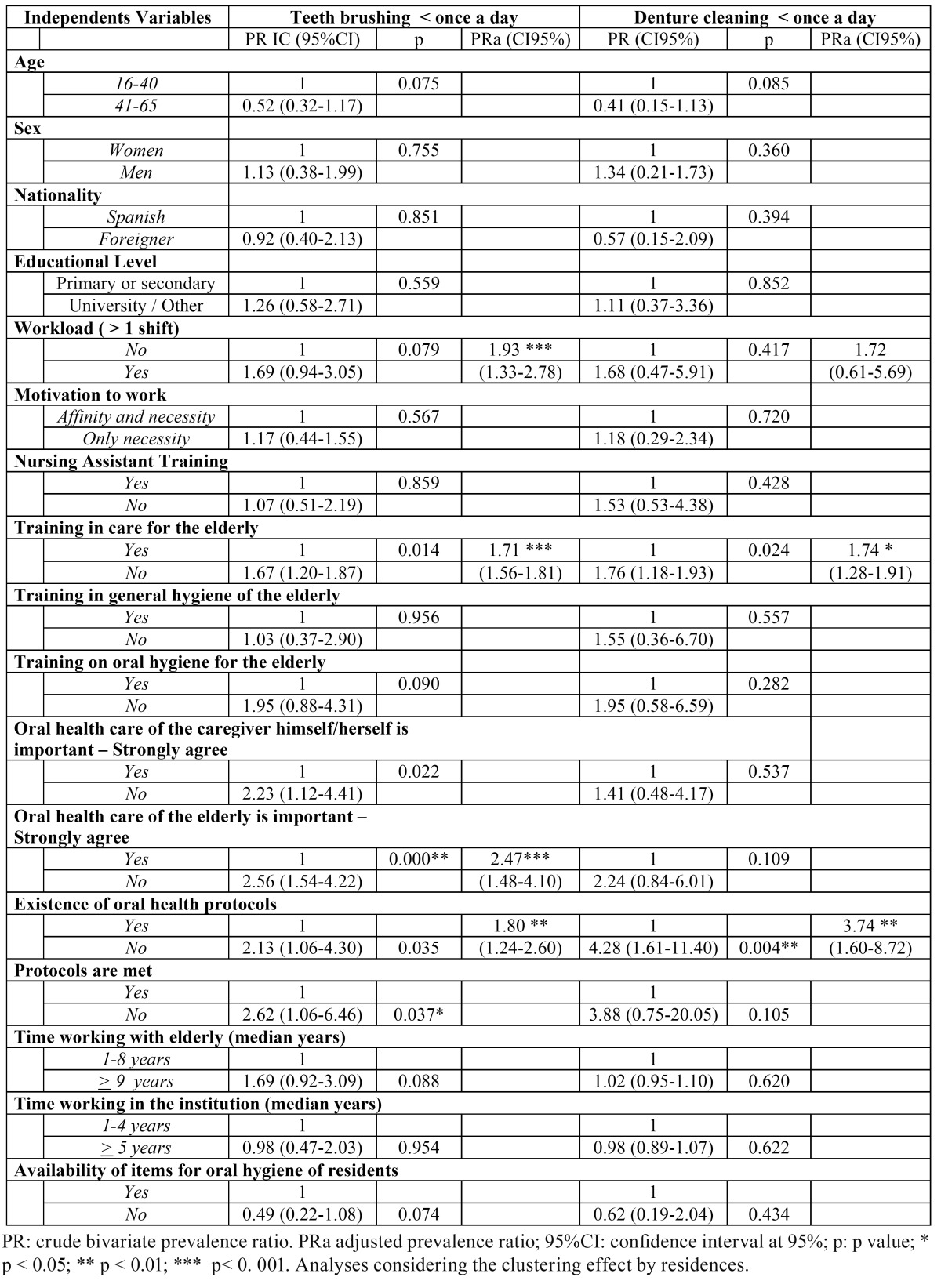
Factors associated with the activities of brushing teeth and cleaning dentures being performed by caregivers less than once a day for people 65 and older institutionalized in long-stay residences, by characteristics of caregivers, Barcelona 2009.

## Discussion

Most caregivers performed activities of oral health care in institutionalized older persons in Barcelona daily, although 11% did so less than once per day, this lower frequency being associated with characteristics of caregivers such as their training and the importance attributed to both their own oral health and that of the elderly, as well as institutional aspects such as the number of hours worked, workload and the existence of protocols on oral health for residents.

The results of this study are of interest considering that institutionalized elderly in Spain have a high prevalence of oral diseases (19,20).

In the task of caring for the oral health of institutionalized older persons, multiple barriers can have a negative impact, including not only individual characteristics of caregivers, but also the social value of oral health, availability of resources, implementation of support policies that also affect institutional attributes which could impact the quality of oral health care provided. Therefore, to overcome these barriers requires a multidisciplinary approach, including the development and implementation of education programs and evaluation of oral health, oral hygiene plans and dental care.

-Characteristics of caregivers: Coinciding with the literature, the caregivers of the study population are mostly women (4,11,21), an aspect that would be attributed to the historical-cultural factor of contemporary society, whereby normally the act of caring falls to women (21). Over 50% have practiced the profession of nursing caregiver for nine or more years, demonstrating extensive experience, even before working on the SHC participating in this study. This is consistent with another study in 169 caregivers of 13 nursing homes, of whom over 50% had experience working in nursing homes (22).

The formal caregivers investigated in this study are people who, in theory, are trained and are responsible for performing a job which requires specific training for the care of institutionalized patients, according to the needs of each elderly resident (23). Proper training of caregivers is critical, as it may either directly or indirectly influence the care of the elderly (10). Urrutia et al. (24) analyzed a sample of 21 formal and 18 informal caregivers of dependent elderly, and observed significant differences between the implementation of some measures of oral health care between the two groups, although they did not find any significant differences between beliefs about oral health by comparing the same groups. The present investigation did not compare formal and informal caregivers. However, coinciding with the literature about the importance of appropriate training of caregivers in oral health (10), in our study the majority of respondents (82,7%) said they had some training as a nursing assistant, but only about 60% had done a formal course, and the caregivers who performed cleaning of dentures more frequently had knowledge or training in nursing care for elderly people. In addition, caregivers who brushed the teeth of elderly patients more frequently had some sort of formal course as nursing assistant or had received specific training in oral hygiene for elderly.

Despite the high prevalence of caregivers with training for general and oral health care of the elderly, according to the literature, it is important that institutions provide updated knowledge on the subject in an efficient manner, in order to ensure the integrity of the health of residents (10). Additionally, the motivation of such institutionalized persons is needed, as their reluctance to receive the oral hygiene and oral health care activities performed by their own caregivers, has been described as an additional barrier to performing oral care and maintaining oral health in the elderly (4,17).

-Prevalence of the activities of oral health care and associated factors: Although there is evidence indicating that brushing teeth after every meal results in better health (15), in the Spanish context in 2009 the related normative documents or protocols whose portfolio of benefits explicitly mentioned the frequency of brushing teeth for institutionalized elderly people in long-stay residential centers, indicated that this should be done at least once per day ( Table 1). In our study, although more than 83% of caregivers performed oral care activities for the elderly at least once a day, those caregivers who performed oral hygiene less often in institutionalized people did not fully agree with the importance of their own oral health, nor that of oral care in elderly people; and 5,6% even answered that oral health care for the elderly was not significant, which may indicate that the attention given to the oral health of the elderly would be seen as secondary. Moreover, considering the evidence, the frequency of performing oral hygiene only once a day is insufficient to maintain oral health. Therefore, these observations provide a warning and indicate the possibility of improving the quality of care, for which it is essential to measure and evaluate the possible improvement activities in order to identify the difficulties that caregivers face in providing this type of care satisfactorily among the elderly (25).

In addition, approximately one third of caregivers reported being motivated to work as a caregiver for the elderly only through necessity. Saliba et al. (in Interface-Comunic, Saúde, Educ 2007;11:39-50) showed that more than half of caregivers of seniors began working through necessity rather than affinity. This finding is of concern, since performing this job without an affinity could compromise the quality of care for the elderly.

In this regard, the literature indicates that perceptions and attitudes of caregivers regarding their own oral health care influence health care activities that they provide for the elderly (4). The fact that the importance of oral care in the elderly is one of the variables explaining the lower frequency of brushing teeth, probably indicates that caregivers believe there is less need for such care in old age, leading to neglect of the care of mouth-parts, essential for the quality of life of the elderly.

-Institutional characteristics: Most caregivers said that the institution had a guide for oral health care for the elderly and those guides were met. The existence of these protocols allows proper guidance for professionals and thus can contribute to improve the oral health of the elderly (14,26). In fact, we note that the existence of protocols proved to be one of the variables that explained the teeth bushing and the cleaning frequency of dentures belonging to the elderly. In centers for the elderly these protocols should be developed considering the material and human resources available and the level of cooperation of patients to establish a workable routine (19). Apart from the reluctance of institutionalized elderly people themselves to allow caregivers to perform oral care for them (4,17), another of the main obstacles to their proper oral care, and with greater social implications, are certain characteristics of the institution, since caregivers must often cope with an excessive work demands due to the high ratio of residents per caregiver and the workload each represents, evidenced in our study by the high percentage of caregivers working more than one shift. Therefore, performing the tasks of oral care for institutionalized elderly people must not be considered a matter of the caregivers’ individual choice.

-Strengths and limitations of the study: This study covered a representative number of nursing assistant caregivers of institutionalized elderly people, since it included 68% of the 46 existing public SHC. The final random sample was 33% of the study population.

Some kind of “expected response” bias could exist, since caregivers were interviewed in the workplace. But in any case, the values showing malpractice are correct, and while 11.7% and 10.2% performed brushing of teeth and cleaning dentures, respectively, less than once day for institutionalized persons, the values may be higher. In this regard, the self-reported frequency of oral health care provided by caregivers for the elderly found in our study and reported in the literature, is in contrast to actual practice observed by Coleman et al. (11). These results could suggest that institutionalized persons often do not receive necessary oral health care (11).

-Recommendations and Conclusions: Oral health care should be, whenever possible, performed by the elderly themselves, be-cause these are activities that exercise motor coordination and raise self-esteem. Our study did not assess the functional capacity of the elderly, but it should be investigated in future studies to determine whether caregivers performed oral hygiene measures in those elderly functionally capable of performing this function themselves. Furthermore, as Coleman et al. (11) reported, it would be important to compare the self-reported frequency of activities provided by caregivers with empirical observation studies.

While the oral health care of older institutionalized people depends on the training received by the caregivers, there are also institutional factors (such as the workload of caregivers and the existence of institutional protocols on oral health care) that may have greater implications for public health. Institutions for the SHC belonging to the Barcelona Health Region should stimulate and improve oral care of the elderly through continuous training and appropriate working conditions of caregivers, and also through the updating of existing protocols on oral health of elderly.
